# Explaining mental health inequalities in Northern Sweden: a decomposition analysis

**DOI:** 10.1080/16549716.2017.1305814

**Published:** 2017-05-31

**Authors:** Nada Amroussia, Per E. Gustafsson, Paola A. Mosquera

**Affiliations:** ^a^ Epidemiology and Global Health, Department of Public Health and Clinical Medicine, Umeå University, Umeå, Sweden

**Keywords:** Mental health, income-related inequalities, concentration index, decomposition analysis, Northern Sweden

## Abstract

**Background**: There has been a substantial increase of income inequalities in Sweden over the last 20 years, which also could be reflected in health inequalities, including mental health inequalities. Despite the growing body of literature focusing on health inequalities in Sweden, income-related inequalities in mental health have received little attention. Particularly scarce are research from Northern Sweden and examinations of the social determinants of health inequalities.

**Objectives**: The present study seeks to provide evidence regarding inequalities in mental health in Northern Sweden. The specific aims were to (1) quantify the income-related inequality in mental health in Northern Sweden, and (2) determine the contribution of social determinants to the inequality.

**Methods**: The study population comprised 25,646 participants of the 2014 Health on Equal Terms survey in the four northernmost counties of Sweden, aged 16 to 84 years old. Income-related inequalities in mental health were quantified by the concentration index and further decomposed by applying Wagstaff-type decomposition analysis.

**Results**: The overall concentration index of mental health in Northern Sweden was

−0.15 (95% CI: −0.17 to −0.13), indicating income inequalities in mental health disfavoring the less affluent population. The decomposition analysis results revealed that socio-economic conditions, including employment status (31%), income (22.6%), and cash margin (14%), made the largest contribution to the pro-rich inequalities in mental health. The second-largest contribution came from demographic factors, mainly age (11.3%) and gender (6%). Psychosocial factors were of smaller importance, with perceived discrimination (8%) and emotional support (3.4%) making moderate contributions to the health inequalities. **Conclusions**: The present study demonstrates substantial income-related mental health inequalities in Northern Sweden, and provides insights into their underpinnings. These findings suggest that addressing the root causes is essential for promoting mental health equity in this region.

## Background

Despite improvements in population health in Europe, health inequities, stemming from inequities in the distribution of resources and money, are increasing substantially between and within the European countries. Tackling health inequities through addressing the social determinants of health has, therefore, become more crucial, in order to enable people to realize their own potentials and to safeguard the future generations’ [1]. In this context, mental disorders are considered part of the main challenges faced by the region in terms of their prevalence, the burden of the disease and the resulting disability [2]. Numerous studies have highlighted the existence of socio-economic inequalities in mental health in European countries [3–6], and reducing mental health inequalities has, therefore, been placed at the core of health policies of Europe and particularly the Nordic countries [2,7].

Although Sweden is considered one of the most equitable Organisation for Economic Co-operation and Development (OECD) countries, income inequalities have increased by one third between 1985 and 2012 as a result of the increase in disparities in market income sources (capital income, self-employment income and gross earnings) and a considerable decline in the redistributive effects of income taxes and cash transfers [8]. This increase in income inequalities represents an urgent threat to the momentous achievements in promoting health and health equity in the country [9]. Mirroring this trend, a growing body of research has revealed an increase of social inequalities in various forms of health in Sweden, including in the large but sparsely populated Northern Sweden [10–12].

The increase in income inequalities in health in Sweden might also be reflected in mental health inequalities; however, this issue has received considerably less attention than, for example, inequalities in cardiovascular disease or self-rated health. This is unfortunate considering the great burden of poor mental health in Sweden as well as its negative impact on people’s quality of life, which involves high costs for the health system and for society in general [13]. The relatively limited body of studies exploring socio-economic inequalities in mental health in Sweden has revealed an inverse association between mental health and economic hardship [10,14,15]. However, to our knowledge, the issue of income-related inequalities in mental health in Northern Sweden has not been explored yet.

Understanding inequalities in mental health from the Northern Swedish perspective may be particularly relevant since the social and public health landscape, as well as the prerequisites for equity in health in the rural and sparsely populated north, differs from the regions of the more populated south. For example, in Sweden the main burden of mild to moderate mental health problems such as anxiety and depression is managed by primary healthcare, which is heavily subsidized and involves a comparatively small patient fee [16,17], whereas severe mental health problems are managed by specialized psychiatric care in hospital [18]. Sweden has a long tradition of tax-financed and publicly provided healthcare, which in international comparisons performs well in terms of costs, distribution of services, and health outcomes [16,17]. All residents are covered by a universal health insurance scheme giving the right to access to subsidized healthcare. Private health insurances play a lesser role than in many other health systems, but exist and can for example guarantee quicker access to consultation or care, or additional payments in case of serious illness [18]. However, the long distances in Northern Sweden involve less accessibility compared to the urban south. Moreover, since the 1990s, the Swedish primary healthcare system has gradually increased its market-orientation [17], most recently exemplified by a 2010 primary healthcare reform allowing all healthcare providers who meet certain basic requirements to establish at a geographical location of their choice [19]. Although the implementation of the reform has resulted in increased new establishments of private healthcare providers in urbanized areas, establishments of private healthcare providers have been proven to be less attractive in the sparsely populated Northern Swedish inland [17], resulting in an even larger regional gap in accessibility of healthcare services, including mental healthcare services. This gap in accessibility is exacerbated by the relative shortage of specialized mental health professionals, such as psychiatrists, in the north compared to the south [20]. Generating context-specific evidence can, therefore, help policy makers to address mental health inequalities as a fundamental step towards reaching one of the main strategic objectives of the current social policies in Sweden [21].

An additional key limitation of international and Swedish research on socio-economic inequalities in health is the dominant focus on describing the existence of the inequalities without identifying their underpinnings, thereby hampering our understanding of the inequalities. Whereas there is a considerable literature on social determinants of *health*, the corresponding social determinants of *health inequalities* are thus not well-understood. Moreover, the majority of studies addressing this question have employed conventional regression models [4,6] which are not well-suited to estimate social inequalities in health or unveil their determinants. During the last few years, decomposition analyses have entered into the public health field as a fruitful approach, not only to quantify the degree of socio-economic-related inequality but also to explain the contribution of each factor to the observed inequality [22]. A few studies have decomposed income inequalities in mental health in Europe [5,23], while, to our knowledge, no such studies have been performed in Sweden.

In order to fill these gaps in the literature, this study aimed to quantify the income-related inequality in mental health in Northern Sweden, and to determine the contribution of a broad range of social determinants of the inequality, using decomposition analysis.

## Methods

### Study population and procedures

Data used for this study were obtained from the 2014 Health on Equal Terms survey of the four northernmost counties in Sweden: Västernorrland, Jämtland/Härjedalen, Västerbotten, and Norrbotten. The survey represents a regionally expanded sample of the smaller sample of the annual National Public Health Survey conducted by the Swedish Public Health Agency in collaboration with county councils/regions and Statistics Sweden [24]. The survey used a two-step probabilistic sampling procedure of all residents aged 16–84 years old in the four northernmost counties, with a response rate of 49.3%. The questionnaire comprised 85 questions covering different topics including health, well-being, consumption of drugs and medicines, health behaviors and social relationships. In addition, socio-demographic data, such as income and education, were linked from the total population registers of Statistics Sweden to the survey through the unique Swedish Personal Identity Number. Data related to education were taken from the education register, while data related to income, economic support and pensions were obtained from the income and taxation register [25].

The total survey sample consisted of 25,667 participants. Due to item non-response, the present study included a maximum of 25,646 participants for complete case analysis.

The use of the Health on Equal Terms survey in the present study was reviewed and approved by the Regional Ethical Review Board in Umeå (approval no. 2015/134–31Ö).

### Measures

#### Outcome variable: G.H.Q-12

Mental health symptoms were assessed through the items from the General Health Questionnaire (GHQ)-12 [24]. The GHQ is considered the most commonly used assessment of mental well-being, and is used to detect common mental health problems such as depression and anxiety [26]. Examples of questions used include ‘Have you been able to concentrate on everything you’ve done in the last few weeks?’, ‘Have you recently been able to deal with your problems?’ and ‘Have you recently felt losing confidence in yourself?’ The respondent answers each item on a four-level Likert scale, and the four response options were then coded as 0; 0; 1; 1. A summative index was first calculated ranging from 0 to 12, and the final binary (dichotomous) variable was created by using a cut-off at ≥ 3 for good (0) vs. poor (1) mental health [24].

#### Socio-economic indicator

The variable used to depict socio-economic status and living standards was individual annual disposable income, which covers income earned from employment, pension, self-employment, and all forms of social benefits (parental leave benefits, long-term disability benefits) as well as negative transfers (e.g. due to debts). The measure thus reflects the amount left for consumption or savings after taxes have been paid and all positive and negative transfers have been made. For calculating the concentration index the variable was used as a continuous variable, and for the decomposition analysis, income was categorized into quintiles with quintile 1 representing the poorest and quintile 5 representing the richest.

#### Social determinants of mental health inequalities

Variables considered as social determinants included *demographic and socio-economic factors* with plausible links to mental health [27]. *Psychosocial factors* that have been associated with poor psychological health and that can possibly contribute to inequalities in poor mental health [27–29] were also included in the analysis. The social determinants’ variables were coded as follows.

*Demographic factors* consisted of four variables: gender was defined as men (0) and women (1). Age was categorized into four groups: 16–29 years (1; young adulthood), 30–44 years (2; early middle age), 45–64 years (3; late middle age) and 65–84 years (4; old age). Civil status was categorized into four groups: married or cohabiting (1), single (2), widowed (3) and divorced (4). Geographic area was represented by the municipality of residence, and categorized into: more than 50,000 inhabitants with hospital (1), 10,000–50,000 inhabitants with hospital (2), 10,000–50,000 inhabitants without hospital (3), and fewer than 10,000 inhabitants without hospital (4).

*Socio-economic factors* cover the socio-economic conditions of the respondents and consisted of four variables. Ethnicity was defined as Swedish (0) and non-Swedish (1). Education was categorized into three groups: low (1; compulsory to < 3 years of secondary education); medium (2; 3 years of secondary to < 3 years of post-secondary education); and high (3; at least 3 years of post-secondary education). Employment status was categorized into six groups: working (1), studying (2), sick pensioner/long-term sick leave (3), unemployed (4), pensioner (5) and other (6). Cash margin was defined as yes (0) when the respondent would be able to gather 15,000 SEK (approx. 1600 EUR) within one week, and no (1) otherwise. The sum of 15,000 SEK was determined by Statistics Sweden and should correspond to a regular worker’s monthly salary [24].

*Psychosocial factors* consisted of three factors: exposure to violence was coded as yes (1) and no (0); emotional support was coded as yes (1) and no (0); and perceived discrimination was coded as yes (1) and no (0).

### Statistical analysis

#### The concentration index

Income-related inequality in mental health was quantified by the concentration index (C) and the concentration curve (CC), using the individual annual disposable income as the socio-economic indicator and the binary GHQ-12 as health outcome. The concentration index is defined as twice the area between the concentration curve and the line of equality (the 45-degree line). The concentration curve is obtained by plotting the cumulative proportion of poor health against the cumulative proportion of the population ranked by the socio-economic indicator. The concentration index can be written as follows [22]:

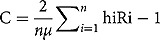

where *h_i_* is the health outcome (GHQ-12: mental health in this study), *μ* is the mean of *h_i_* and *n* the number of people. *R_i_* represents the fractional rank of individual *i* in the living standards distribution used (the annual personal income), with *i* taking the value of 1 for the poorest and the value of *n* for the richest [22].

If the curve lies above the line of equality, the concentration index takes a negative value, indicating a disproportionate concentration of inequality among the poor (pro-rich). Conversely, if the curve lies below the line of equality, the concentration index takes a positive value, indicating a disproportional concentration of inequality among the rich (pro-poor) [22]. In the absence of socio-economic-related inequality, the concentration index is zero.

As the outcome variable in the present study is binary, the bounds of C do not vary between 1 and −1 but rather depend on the mean (*µ*) of the variable. Thus the bounds of C vary between *µ*–1 (lower bound) and 1–*µ* (upper bound) and the interval shrinks when the mean (*µ*) increases [22]. As a correction, the present study applied the Wagstaff normalization to calculate the concentration index by dividing C by 1 minus the mean (1–*µ*).

#### Decomposition of the concentration index

To determine the contribution of each social determinant to the observed health inequalities, a decomposition analysis of the concentration index was used. The overall concentration index can be decomposed into contributions of *k* social determinants, in which each social determinant’s contribution is obtained by multiplying the sensitivity of the outcome variable with respect to that determinant and the degree of income-related inequality in that factor.

Based on a linear additive regression model, the concentration index for mental health (*y*) can be expressed as follows [22]:

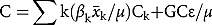

where *µ* is the mean of *y*, 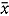
_k_ is the mean of x_k,_ C_k_ is the concentration index of x_k_, and GC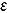 (residual) is the generalized concentration index for the error term (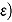. The overall concentration index of mental health (*y*) includes an explained part formed by the sum of the contributions of *k* determinants, and an unexplained part (residual) [22]. The sum of the contributions of *k* determinants is equal to the weighted sum of the concentration indices of the determinants, multiplied by the weights of x_k_ (η_k_ = β_k_ 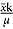) which is the elasticity of *y* with respect to each *x*_k_.

Based on the the Wagstaff normalization [22], the normalized decomposition of the concentration index, obtained by dividing the concentration index by 1–*µ*, can be rewritten as follows:

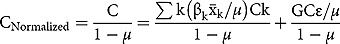

To handle the binary outcome, the non-linear estimation method proposed by Wagstaff [22] was used to restore the underlying assumptions of the decomposition method. First, the marginal effects of the β_k_ were calculated using a probit model. Then, these marginal effects were used to calculate the contributions of the *k* determinants. A linear approximation of the non-linear estimations can be expressed as follows:

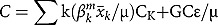

A social determinant can contribute to inequality in mental health in one of these two cases: first, if the determinant has a high prevalence among people with low income and was associated with high probability of reporting poor mental health. Second, if the determinant has a high prevalence among people with high income and was associated with low probability of reporting poor mental health [30].

## Results

### Characteristics of the study population

Characteristics of the study population including the prevalence of poor mental health by explanatory variable are outlined in Table 1. About half (53.8%) of the study sample were women, and two thirds (64.9%) were below 65 years old. Most of the participants were Swedish (93.4%), married or cohabiting (69%), had completed compulsory education (50.1%), were working (45.1%), and resided in rural municipalities with fewer than 10,000 inhabitants (39.3%). One sixth (16.6%) of the participants reported no low cash margin, and 10.8% lacked emotional support. Only 4% of the participants reported being exposed to violence although 15.7% had experienced discrimination.

**Table 1.** Descriptive statistics of the study population and proportions of poor mental health.

	Variable frequencies		Prevalence of poor mental health	
	N	%	N	%
**GHQ-12**				
Good	22,084	86.1	3562	13.9
**Sex**				
Men	1184	46.2	1319	11.1
Women	13,806	53.8	2243	16.3
**Age**				
16–29 years	4173	16.3	877	21.0
30–44 years	5072	19.8	905	17.8
45–64 years	739	28.8	972	13.2
65–84 years	9011	35.1	808	9.0
**Education**				
Low	12,532	50.1	1571	12.5
Medium	8311	33.3	1228	14.8
High	4154	16.6	636	15.3
**Civil status**				
Married/cohabiting	17,685	69.0	2188	12.4
Single	5054	19.7	991	19.6
Divorced	1555	6.1	240	15.4
Widower	1352	5.3	143	10.6
**Ethnicity**				
Swedish	23,955	93.4	3273	13.7
Non-Swedish	1691	6.6	289	17.1
**Municipality of residence**				
More than 50,000 inhabitants with hospital	6231	24.3	935	15.0
10,000–50,000 inhabitants with hospital	4928	19.2	618	12.5
10,000–50,000 inhabitants without hospital	4407	17.2	653	14.8
Fewer than 10,000 inhabitants without hospital	1008	39.3	1356	13.5
**Income (quintile)**				
1 (poorest)	5113	20.0	959	18.8
2	5113	20.0	750	14.7
3	5113	20.0	707	13.8
4	5113	20.0	656	12.8
5 (richest)	5113	20.0	471	9.2
**Cash margin**				
Yes	21,204	83.4	2431	11.5
No	4217	16.6	1099	26.1
**Employment status**				
Working	11,561	45.1	1385	12.0
Studying	1943	7.6	438	22.5
Sick pensioner/long-term sick leave	1168	4.6	402	34.4
Unemployed	967	3.8	257	26.6
Pensioner	7239	28.2	640	8.8
Other	2768	10.8	440	15.9
**Violence**				
Yes	1021	4.0	334	32.7
No	24,625	96.0	3228	13.1
**Emotional support**				
Yes	22,244	89.2	2702	12.2
No	2692	10.8	745	27.7
**Perceived discrimination**				
Yes	4026	15.7	1338	33.2
No	2162	84.3	2224	10.3

The overall prevalence of poor mental health among the study participants was 13.9%, higher among women (16.3%), those who are non-Swedish (17.1%) and the youngest participants (21%). The prevalence of poor mental health was particularly high among participants who were exposed to violence (32.7%) or who experienced discrimination (33.2%). Mental health displayed a clear income gradient, with the highest prevalence found among people within the lowest quintile (18.8%), and the lowest in the highest quintile (9.2%).

### Income-related inequality in mental health

**Figure 1.**
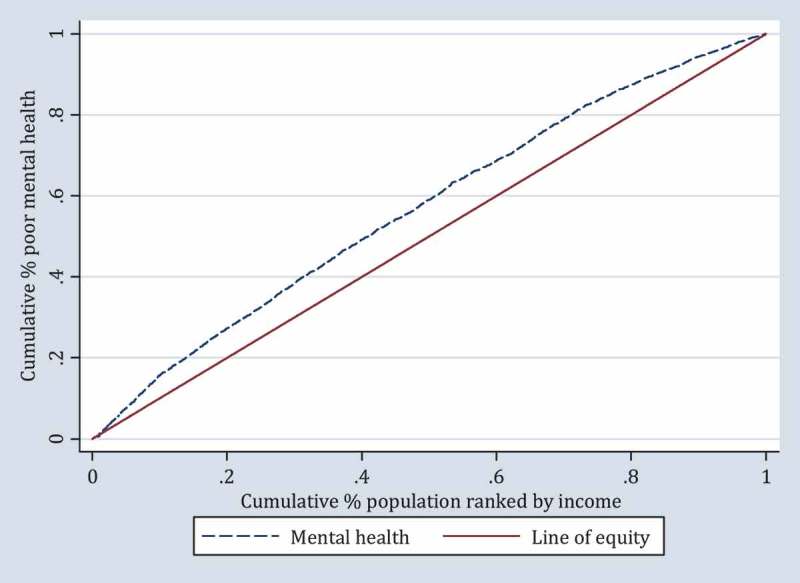
Concentration curve (C = −0.15, 95% C.I: −0.17 to −0.13).

The concentration curve of income-related inequality in mental health is shown in Figure 1. The concentration curve lay above the diagonal line of equity, with the overall concentration index estimated at −0.15 (95% CI: −0.17 to −0.13), thus indicating pro-rich health inequalities, meaning that poor mental health was concentrated among people with lower income.

The results of the decomposition analysis are presented in Table 2. The analysis revealed that socio-economic and material conditions such as employment status (31% contribution to the explained part of the inequalities), income (22.6%) and cash margin (14%) played the largest role in explaining the pro-rich inequalities in mental health. Demographic factors came next in independent explanatory importance, with age (11.3%) and gender (6%) being the most important contributors among this group of variables. Psychosocial factors were third in importance, with perceived discrimination (8%) and emotional support (3.4%) making small to moderate contributions to the inequalities affecting less affluent people. Finally, education, municipality of residence, exposure to violence and civil status made insubstantial independent contributions (equal or less than 2% contribution) to the observed inequality.

**Table 2.** Summary of results of decomposition analysis.

	Coeff.	Elast.	C	Cont. to C	%	Adjusted % contribution
**Sex**						
Men						
Women	**0.03**	0.291	−0.038	−0.011	7.4	6.0
**Age**						
16–29 years						
30–44 years	0.000	0.001	0.219	0.000	−0.1	
45–64 years	**−0.034**	−0.071	0.295	−0.021	13.9	11.3
65–84 years	**−0.072**	−0.183	−0.128	0.023	−15.6	
**Education**						
Low	**−0.032**	−0.115	−0.148	0.017	−11.4	
Medium	**−0.019**	−0.046	0.080	−0.004	2.4	2.0
High						
**Civil status**						
Married/cohabiting						
Single	−0.009	−0.013	−0.333	0.004	−2.8	
Divorced	0.007	0.003	0.013	0.000	0.0	
Widower	0.006	0.002	−0.160	0.000	0.2	
**Ethnicity**						
Swedish						
Non-Swedish	−0.011	−0.081	−0.010	0.001	−0.5	
**Municipality of residence**						
More than 50,000 inhabitants with hospital						
10,000–50,000 inhabitants with hospital	**−0.022**	−0.030	0.049	−0.001	1.0	0.8
10,000–50,000 inhabitants without hospital	−0.005	−0.006	−0.009	0.000	0.0	
Fewer than 10,000 inhabitants without hospital	**−0.017**	−0.049	−0.053	0.003	−1.7	
**Income (quintile)**						
1	**0.025**	0.035	−0.798	−0.028	18.8	15.3
2	**0.024**	0.034	−0.399	−0.014	9.0	7.3
3	**0.021**	0.030	−1.5E-09	0.000	0.0	
4	*0.017*	0.025	0.399	0.010	−6.6	
5						
**Cash margin**						
Yes						
No	**0.065**	0.078	−0.333	−0.026	17.3	14.0
**Employment status**						
Working						
Studying	**0.047**	0.025	−0.664	−0.017	11.3	9.2
Sick pensioner/long-term sick leave	**0.202**	0.066	−0.173	−0.011	7.6	6.2
Unemployed	**0.084**	0.023	−0.371	−0.008	5.7	4.6
Pensioner	**0.044**	0.090	−0.137	−0.012	8.3	6.7
Other	**0.044**	0.034	−0.231	−0.008	5.3	4.3
**Violence**						
Yes	**0.049**	0.014	−0.080	−0.001	0.8	0.6
No						
**Emotional support**						
Yes						
No	**0.089**	0.711	−0.009	−0.006	4.2	3.4
**Perceived discrimination**						
Yes	**0.152**	0.172	−0.086	−0.015	9.9	8.0
No						
**Concentration index (C)**				−0.150		
**Standard error (s.e)**				0.010		
**Residual**				−0.024		
**95% confidence interval (C.I)**	(−0.170 to −0.130)					

Notes: **Bold** indicates *p* < 0.01, *italics* indicate 0.01 ≤ *p* < 0.05. **Coeff**.: coefficient; **Elast**.: elasticity; **C**: concentration index; **Cont. to C**: contribution to concentration index; **%**: percentage contribution.

Table 2 shows additional information useful to understand these contributions. As indicated by the negative concentration indices (C), women, students, the unemployed, sick pensioners, people without cash margins, people lacking emotional support and people exposed to perceived discrimination were more concentrated among the less affluent population. These groups contributed to the inequalities in mental health as they simultaneously showed higher probabilities of reporting poor mental health, as indicated by the coefficients (Coeff.).

The residual reflecting the non-explained part of inequality in mental health was very small (0.024), indicating that the social determinants included in the model jointly explained a considerable part of the estimated inequality of mental health in Northern Sweden (86%).

## Discussion

To our knowledge, this is the first study decomposing income-related inequalities in mental health in Sweden. The study revealed substantial pro-rich income-related inequalities. The decomposition results suggest that these inequalities to a considerable degree are underpinned by socio-economic factors (employment status, income and cash margin), with demographic factors (age and gender) and psychosocial factors (perceived discrimination, lack of emotional support and violence) also contributing to the inequality.

The findings of the present study are generally in line with other studies decomposing income inequalities in mental health, in Europe [5,23], North America [31] and in low- and middle-income countries [32–34], pointing out the existence of inequalities disfavoring the less affluent population. Although it is hazardous to make a straight comparison between the Cs, it should be noted that the magnitude of inequalities in mental health in the present study is larger than that reported in a former study conducted in Europe [23]. This surprisingly large magnitude of income inequalities in mental health in Northern Sweden could possibly be a reflection of the substantial increase in income inequalities in Sweden, coupled with the strong impact of economic hardship on mental health previously reported in the country [13,35].

We furthermore found that employment status was the factor making the largest independent contribution to the mental health inequalities, whereas education played a lesser role. Another study conducted in the Nordic countries [36] instead reported an important contribution of occupation class and education to income inequalities in physical and mental self-reported health. However, that particular study did not include any other explanatory factors, and the set of explanatory factors was thus much more limited than in the present study. The substantial contribution of being a sick pensioner or benefiting from long-term sick leave found in the present study might be explained by the fact that mental illness represents the lead cause for long-term sick leave and disability in Sweden [37], and that long-term sick leave is much more common among those with lower income [38].

Income inequalities themselves accounted for a sizeable contribution to the inequalities in mental health. Whereas previous studies are inconsistent regarding the association between income and poor mental health in Sweden [35,39], our findings thus point to a substantial association between income and not only mental health outcomes but also mental health inequalities. Moreover, several former studies in Sweden have also revealed that people suffering from economic problems such as limited cash margin have a higher prevalence of poor mental health including anxiety and depression [13,35,40]. While the previous studies focused only on the association between the lack of cash margin and poor mental health, the present study adds to the literature by highlighting the contribution of this factor to mental health inequality.

This study also suggests that other demographic and psychosocial factors play an important role in explaining mental health inequalities. For example, women were contributing to the inequality in mental health, as they have worse mental health and also lower income, which is in line with previous research from Sweden including Northern Sweden [13,41]. It is noteworthy that despite the national policies promoting gender equality in Sweden, inequalities in the labor market and in salaries are still persistent [42]. Here, our findings exemplify the complex role of gender when it comes to explaining income-related inequalities in health, and also mirror the worrying increase of inequalities in other health outcomes and life expectancy affecting socio-economically disadvantaged women [43,44].

One of the interesting findings of this study is that the contributions of the youngest and oldest groups were offsetting the inequality. These findings diverge from previous studies conducted in other high- and middle-income countries [23,33], suggesting that old age is a strong contributor to income inequalities in mental health. However, our findings are consistent with a former study performed in Sweden showing that people aged over 65 reported better mental health compared to young people [13]. This might be explained by the fact that stress related to financial strain in middle age decreases in older age [45]. Moreover, due to the well-established pension system in Sweden, elderly people usually benefit from welfare policies that ensure adequate economic support and health insurance, which might be reflected positively in their mental health [46].

Among psychosocial factors, perceived discrimination and lack of emotional support contributed moderately to the inequalities in mental health. Although the associations between these psychosocial factors and poor mental health [27] and socio-economic status [29,47] have been extensively explored, to the authors’ knowledge, these factors have seldom been specifically examined as underpinning socio-economic inequalities in mental health, and never by decomposition analysis. Previous studies have, for example, pointed out that perceived discrimination, one of the strongest contributors to the inequalities in our study, is associated with poor mental health [28] and more frequent among low socio-economic positions [48], and that socio-economic disadvantage partially explains the association between perceived discrimination and psychological distress in Sweden [49]. Similarly, lack of emotional support [50] and exposure to violence [29] are established predictors of poor mental health, and are also more common among those of a lower socio-economic position [51,52].

Taken together, the present study thus brings together the previous piecemeal evidence by showing the independent contributions of these psychosocial factors to income-related inequalities in mental health in Northern Sweden. These results would suggest that addressing inequalities in mental health requires comprehensive interventions to tackle both material and psychosocial factors.

### Methodological considerations

The main strengths of this study include a large population-based sample, combination of survey data and national register data, and the use of decomposition analysis, an appropriate method to estimate and explain health inequalities. Nevertheless, the present study has several limitations.

A major limitation is the cross-sectional design, which does not allow for causal inference. Additionally, the low response rate of the survey (49.3%) invites potential selection bias in the study population, which could have reduced the internal and external validity of the present study. For example, people with severe mental health problems could have been underrepresented in the study population, yielding potentially biased estimates of GHQ scores and the prevalence of poor mental health. Nevertheless, this selection bias might have a minor effect on the point estimates of the present study, the concentration index and the decomposition estimates, unless the non-participants also differ substantially from the participants when it comes to the concentration of mental health along the income range.

Concerning the measures, income has been established as a major social determinant of mental health and a common measure of people’s socio-economic status [5,14]. Nevertheless, disposable income does not fully reflect the financial situation of an individual, e.g. income from other household members or wealth, although the latter is partially tapped into by cash margin.

Although our model explained most of the income inequalities in mental health in Northern Sweden, unmeasured social determinants accounted for 16% of the observed inequality. Due to the lack of available data, other potential explanatory factors such as household size, number of children at the household and private health insurance were not included in this model. We applied the Wagstaff normalization to the concentration index and the decomposition to handle the use of a binary outcome with a method that relies on linear regression. However, it should be noted that other possible corrections could have been used and would have resulted in different inferences [53].

## Conclusions

This study provides evidence of considerable income-related inequalities in mental health in Northern Sweden disfavoring the less affluent population. Findings suggest that the greater part of this inequality can be attributed to socio-economic, material, demographic and psychosocial factors. The results thus suggest that without addressing the root causes of socio-economic inequalities, including income and employment inequalities as well as the structural disadvantage of women, health inequalities in mental health may endure in the population of Northern Sweden.

## Paper context

There has been a substantial increase of income inequalities in Sweden over the last 20 years, which might be reflected in health inequalities, including mental health inequalities. This paper tries to provide context-specific evidence regarding income-related inequalities in mental health in Northern Sweden, using decomposition analysis to identify the underpinnings of the inequalities. Comprehensive strategies to tackle socio-economic factors and psychosocial factors are needed in order to address these inequalities.
